# Genotype- and rootstock-mediated adaptation of fruit trees to climate change

**DOI:** 10.1186/s40659-026-00720-y

**Published:** 2026-08-12

**Authors:** Hamid Reza Soufi, Mani Jabbari, Yazgan Tunç, Mitra Jabbari, Ali Khadivi, Mansour Ghorbanpour

**Affiliations:** 1 Department of Horticultural Sciences, Faculty of Agriculture, Vali-e-Asr University of Rafsanjan, Rafsanjan, Iran; 2 Department of Horticultural Science and Engineering, Faculty of Agriculture, Birjand University, Birjand, Iran; 3 Republic of Türkiye Ministry of Agriculture and Forestry, General Directorate of Agricultural Research and Policies, Hatay Olive Research Institute Directorate, Hassa Station, 31700 Hassa, Hatay Türkiye; 4 Department of Horticultural Science and Engineering, Faculty of Plant Production, Gorgan University of Agricultural Sciences and Natural Resources, Gorgan, Iran; 5 Department of Horticultural Sciences, Faculty of Agriculture and Natural Resources, Arak University, Arak, 38156-8-8349 Iran; 6 Department of Medicinal Plants, Faculty of Agriculture and Natural Resources, Arak University, Arak, 38156-8-8349 Iran

**Keywords:** Climate change, Phenology of fruit trees, Rootstock–scion interaction, Genotype adaptation, Abiotic stress

## Abstract

Climate change is increasingly threatening the sustainability and productivity of fruit tree systems worldwide. Rising temperatures, altered precipitation patterns, more frequent extreme weather events, and elevated atmospheric CO_2_ are affecting physiological and phenological processes that regulate tree growth, reproduction, and fruit quality. Key impacts include reduced winter chilling, disrupted dormancy release, irregular flowering, impaired pollination, and altered fruit development, leading to lower yield stability and quality. Due to their long life cycles, fruit trees are particularly vulnerable to cumulative environmental stresses such as heat, drought, salinity, and irregular rainfall, which can impair photosynthesis, growth, and reproductive performance while increasing susceptibility to pests and diseases. This review synthesizes current knowledge on the effects of major climate-related factors, including temperature, water availability, and elevated CO_2_, on the physiology, phenology, pollination, and productivity of fruit trees. Particular emphasis is placed on the role of genotype diversity and rootstock–scion interactions in improving climate resilience. The review also highlights key adaptation strategies, including the use of climate-resilient cultivars, optimized rootstock selection, efficient water and nutrient management, and climate-smart orchard practices. By integrating physiological, ecological, and production-level responses, this review provides a framework for enhancing the resilience and sustainability of fruit production under changing climatic conditions.

## Introduction

**Fig. 1:**
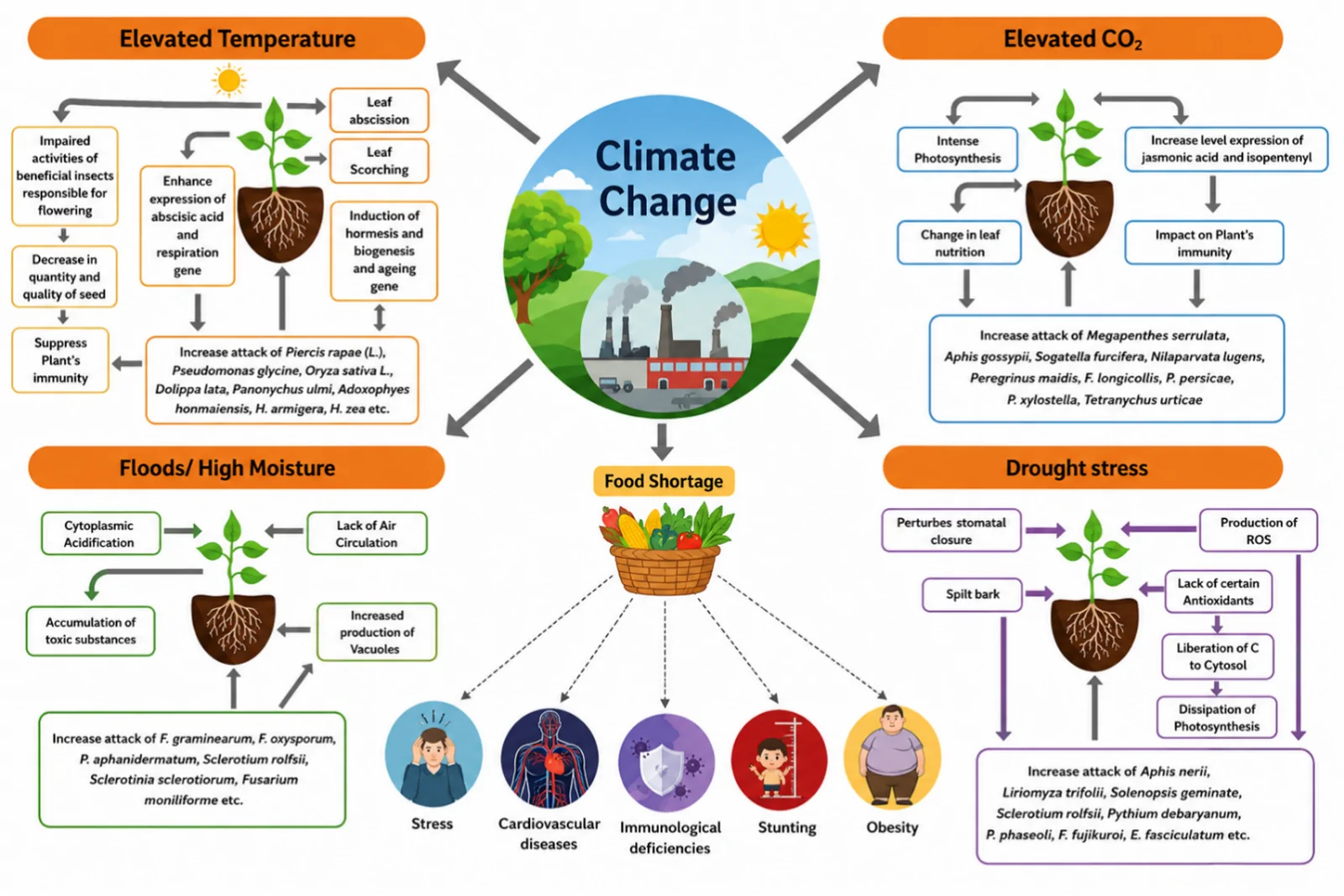
Conceptual framework illustrating the cascading impacts of climate change on plant health, food security, and human health (taken from [98], with the author's permission)

Climate change is a major global challenge that is increasingly threatening agricultural sustainability, particularly perennial fruit production. Rising temperatures, erratic rainfall, more frequent droughts, floods, heat waves, and hailstorms are already reducing fruit yield and quality [88, 217]. According to the Sixth Assessment Report of the IPCC, global surface temperatures are projected to increase by approximately 2.5–4 °C by the end of this century under many emission scenarios, while atmospheric CO_2_ concentrations may reach 550–800 ppm, intensifying the greenhouse effect and climate instability [127]. These climatic changes disrupt all stages of fruit tree development, from dormancy release and flowering to fruit growth, ripening, and leaf senescence [142]. Anthropogenic climate change results from long-term alterations in atmospheric conditions caused primarily by greenhouse gas emissions from fossil fuel combustion, deforestation, industrialization, and land-use change [97]. Increasing concentrations of CO_2_, methane, nitrous oxide, and ozone have accelerated global warming, leading to more frequent extreme weather events, including heat waves, droughts, frosts, floods, and irregular precipitation [152]. Agriculture is among the sectors most vulnerable to these changes because climate determines crop distribution, productivity, and pest and disease dynamics [218]. Fruit trees are particularly susceptible because, as perennial and sessile plants, they remain exposed to repeated environmental stresses throughout their lifespan. Climatic disturbances have already been linked to irregular budbreak, altered flowering phenology, uneven fruit development, and reduced productivity [142]. Future warming may reduce crop yields by 30–80% depending on species and stress intensity, highlighting the urgent need for adaptation strategies beyond conventional breeding [200]. Integrating climate-resilient cultivars with improved orchard management practices will therefore be essential for sustaining fruit production under future climate scenarios (Fig. 1).

**Fig. 2:**
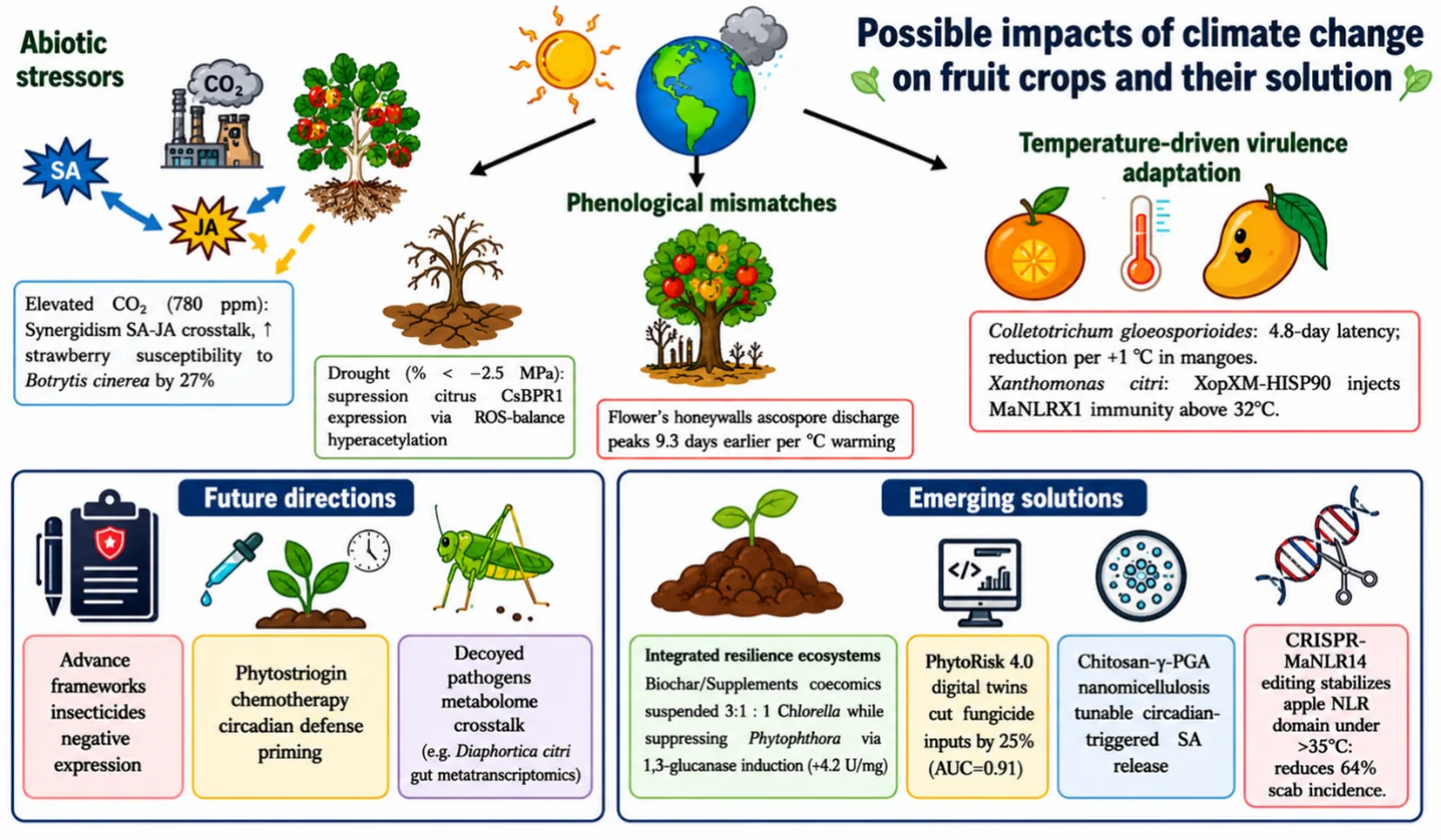
Summary of the major pathways through which climate change affects fruit production, including temperature rise, altered precipitation regimes, and elevated atmospheric CO_2_ (taken from [98], with the author's permission)

**Fig. 3:**
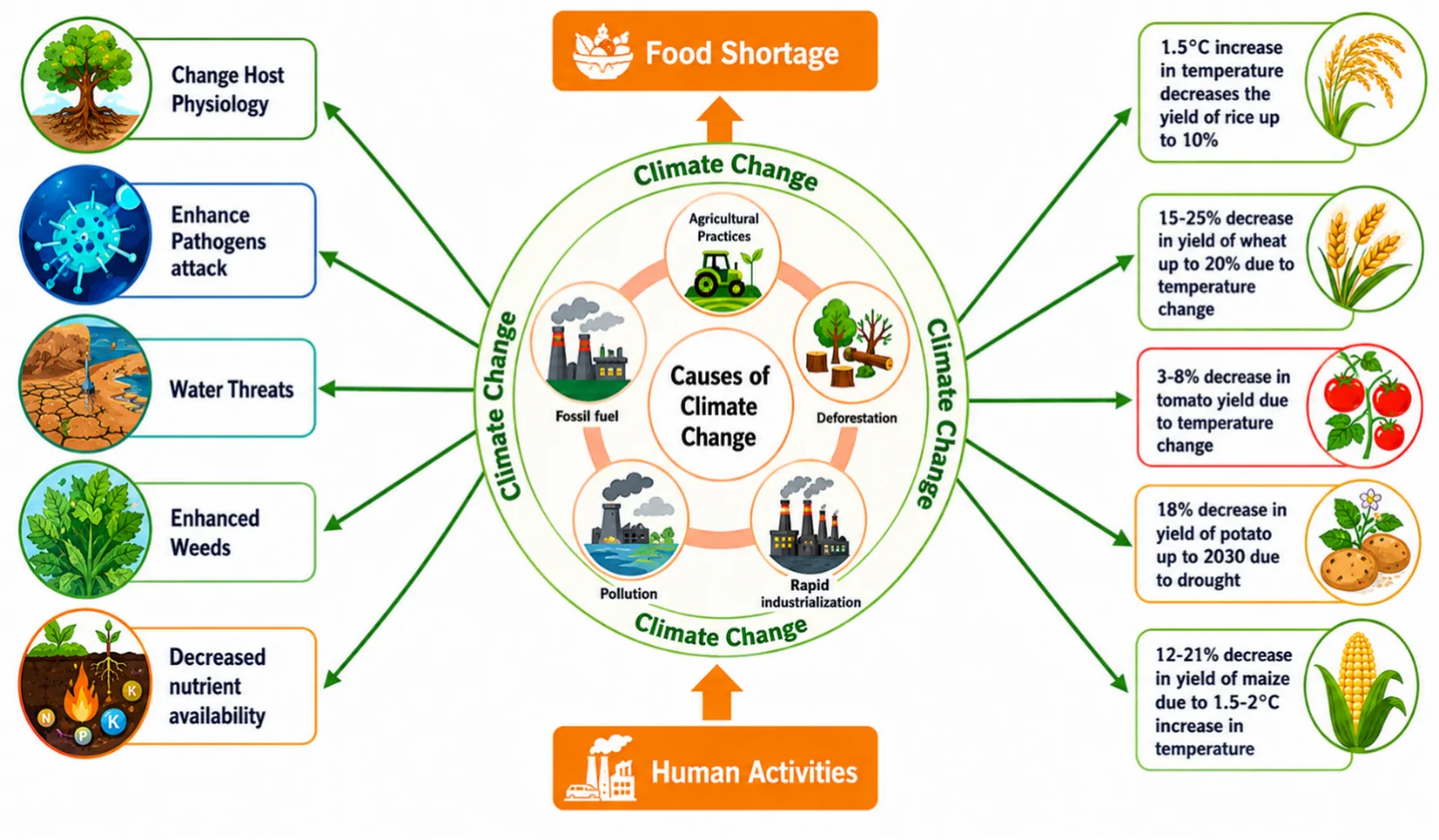
Cause–effect schematic linking key drivers of climate change (e.g., industrialization and deforestation) to downstream impacts on food production, including yield losses mediated by warming, drought, and water scarcity (taken from [98], with the author's permission)

**Fig. 4:**
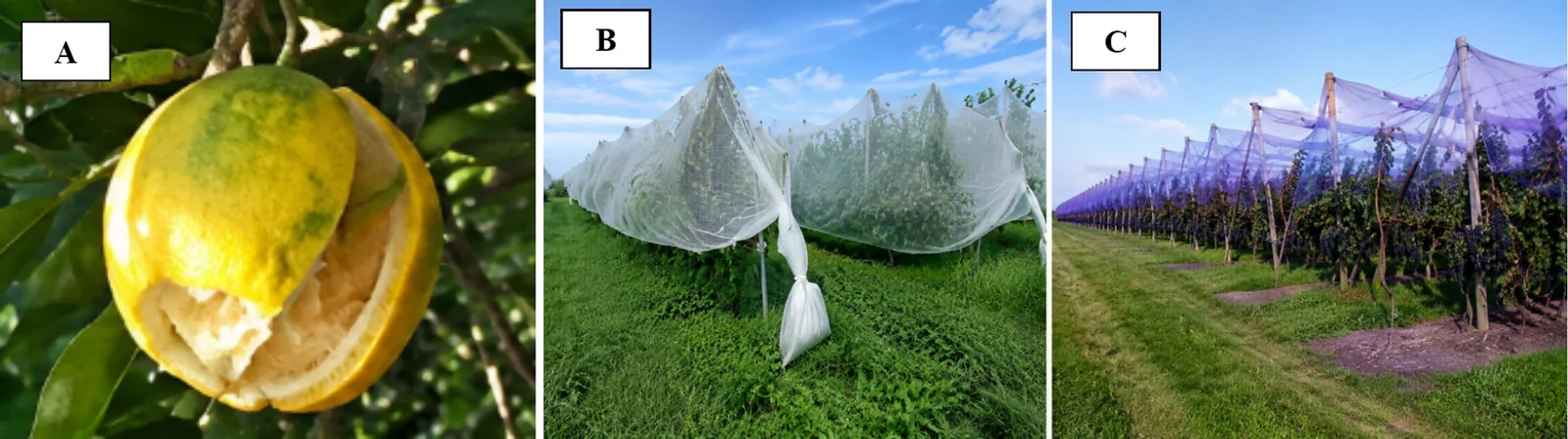
Examples of climate-related physiological disorders and protective management in fruit crops: **A** fruit cracking in Valencia orange (taken from [1], with the author's permission); **B** and **C** protective orchard roof netting used to reduce frost injury risk

Rising temperatures are among the most significant consequences of climate change, posing serious threats to fruit production across temperate, tropical, and subtropical regions (Fig. 2). In addition to warming, changes in atmospheric CO_2_ concentration and rainfall patterns strongly influence orchard productivity and management [150]. These climatic changes reduce water availability, increase irrigation requirements, alter harvest timing, increase pest pressure and physiological disorders, and accelerate soil degradation, ultimately decreasing fruit quality and limiting the availability of climate-adapted cultivars (Fig. 3) [150]. Fruit species differ in their optimum temperature requirements; therefore, even modest warming can shift suitable production areas and reduce productivity. Elevated temperatures disrupt flowering, fruit set, and dormancy regulation, while prolonged heat and low humidity increase physiological disorders such as sunburn and fruit cracking in apple, apricot, and cherry, and fruit splitting in litchi (Fig. 4A–C) [121]. Heat stress also accelerates fruit maturation, shortening harvest periods and negatively affecting sugar accumulation, organic acid metabolism, pigment formation, antioxidant content, peel coloration, texture, and overall fruit quality [119].

Fruit production is strongly dependent on climatic conditions, making orchards particularly vulnerable to climate variability. Changes in temperature, atmospheric CO_2_, precipitation, water availability, pollination, and pest and disease pressure directly influence tree growth, fruit development, yield, and quality [150]. Climate-induced disruptions affect phenology, irrigation demand, orchard management, fruit composition, and postharvest performance, threatening the sustainability of fruit production systems. Therefore, effective adaptation and mitigation strategies are essential to maintain productivity and ecosystem resilience under future climatic conditions. Despite the increasing number of studies on climate change and horticulture, most reviews have focused on individual stress factors such as heat, drought, or phenological shifts, with limited integration of physiological, ecological, and production responses. Moreover, previous studies have emphasized annual crops, whereas perennial fruit trees present unique challenges because of their long lifespan, complex phenology, and chilling requirements. The interactions among dormancy regulation, pollination, genotype adaptability, and long-term orchard sustainability under changing climates remain insufficiently synthesized. This review addresses these knowledge gaps by integrating current evidence on the effects of temperature, precipitation, elevated CO_2_, and associated biotic stresses on fruit trees. It also identifies key vulnerability thresholds and summarizes adaptation strategies for developing climate-resilient and sustainable fruit production systems.

## Literature search and review methodology

This review was conducted as a narrative synthesis of the current scientific literature on genotype- and rootstock-mediated adaptation of fruit trees to climate change. To ensure comprehensive coverage, relevant publications were identified through systematic searches of major international bibliographic databases, including Web of Science Core Collection, Scopus, ScienceDirect, SpringerLink, PubMed, Wiley Online Library, Taylor & Francis Online, MDPI, Frontiers, and Google Scholar. The literature search primarily covered publications from 2000 to 2026, although earlier landmark studies were included when they provided essential background or established fundamental concepts related to rootstock–scion interactions, fruit tree physiology, or climate adaptation. Searches were performed using combinations of keywords connected with Boolean operators (AND/OR), including climate change, global warming, fruit trees, orchard, genotype, cultivar, rootstock, scion, rootstock–scion interaction, grafting, drought stress, heat stress, salinity, waterlogging, flooding, elevated CO_2_, winter chilling, flowering, phenology, photosynthesis, water-use efficiency, nutrient uptake, abiotic stress, climate resilience, stress tolerance, precision orchard management, and fruit quality. Additional relevant publications were identified through citation tracking of key review articles and highly cited original research papers. Only peer-reviewed research articles and authoritative review papers published in English were considered. Preference was given to studies involving commercially important temperate, subtropical, and Mediterranean fruit tree species that investigated genotype-dependent responses, rootstock effects, rootstock–scion interactions, physiological and molecular mechanisms of stress adaptation, or orchard management strategies under climate-related abiotic stresses. Studies lacking sufficient experimental detail, publications unrelated to perennial fruit crops, conference abstracts, non-peer-reviewed reports, and duplicate records were excluded. The collected literature was critically synthesized rather than summarized descriptively. Particular emphasis was placed on identifying common physiological, molecular, and agronomic mechanisms through which genetic variation, rootstock selection, and rootstock–scion interactions contribute to adaptation under drought, heat, salinity, flooding, elevated atmospheric CO_2_, and insufficient winter chilling. The evidence was integrated across fruit species to identify consistent patterns, knowledge gaps, and future research priorities for developing climate-resilient fruit production systems.

## Morphophysiological and biochemical responses of fruit trees to climate change

### Impact of climate change on plant growth

In some high-latitude regions, moderate warming may extend the growing season and stimulate vegetative growth. However, these benefits depend on adequate water availability because rising temperatures are frequently accompanied by reduced precipitation and greater drought stress [186]. In fruit trees bearing fruit on one-year-old shoots, such as peach and olive, restricted shoot growth reduces flower bud formation and lowers yield in the following season. Excessive summer heat also causes leaf injury, suppressing photosynthesis, transpiration, and assimilate production required for bud differentiation and fruit development [186]. Consequently, heat and water deficits disrupt key developmental processes, including dormancy, flowering, fruit set, and ripening [68, 99]. Elevated atmospheric CO_2_ can partially offset some adverse climatic effects by enhancing photosynthesis through the CO_2_ fertilization effect. Increasing atmospheric CO_2_ concentrations from approximately 400 ppm to 600–750 ppm can increase biomass production of many C_3_ species by about 30%, provided that water, nutrients, and vapor pressure deficit remain non-limiting [246]. In fruit trees, elevated CO_2_ has been associated with greater shoot growth, fruit yield, fruit size, and sugar accumulation, although fruit nutritional quality may decline because of mineral dilution [69]. For example, long-term CO_2_ enrichment increased citrus biomass by nearly 70% through enhanced branching and fruit production [115]. Nevertheless, prolonged exposure often induces photosynthetic acclimation as carbohydrate accumulation, restricted sink capacity, and nutrient limitations progressively reduce photosynthetic efficiency [86]. Elevated CO_2_ also modifies canopy architecture and plant morphology. Increased leaf area, shoot elongation, root growth, branching, and hydraulic development are common responses, although their magnitude varies among species and cultivars [128]. Although reduced stomatal aperture generally improves water-use efficiency, larger canopies may offset these gains by increasing total transpiration. Hormonal regulation is also altered, with elevated CO_2_ affecting auxins, cytokinins, gibberellins, abscisic acid, and jasmonic acid, thereby influencing vegetative growth and drought responses [128]. Excessive vegetative vigor may delay flowering, increase pruning requirements, and reduce orchard efficiency. The magnitude of the CO_2_ fertilization effect is strongly influenced by water and nutrient availability, particularly nitrogen and phosphorus [137]. While elevated CO_2_ can improve water-use efficiency under moderate drought, its benefits decline under severe heat or nutrient limitation. Long-term CO_2_ enrichment frequently reduces tissue concentrations of essential macro- and micronutrients, including N, P, Ca, K, Mg, Fe, Zn, and Mn, primarily because of biomass dilution and reduced nutrient transport [69]. Consequently, sustaining fruit productivity under future climates will require precise nutrient management strategies that optimize fertilizer use while maintaining fruit quality [179]. Climate change will also alter soil nutrient cycling through changes in mineralization, decomposition, nutrient leaching, and nutrient availability [62]. Because responses to elevated CO_2_ differ among cultivars, rootstocks, and orchard planting systems, future productivity will depend on selecting genotypes capable of efficiently utilizing water, nutrients, and assimilates under changing climatic conditions [137]. Integrating climate-resilient cultivars with sustainable orchard and nutrient management will therefore be essential to maintain long-term fruit production. Recent studies demonstrate that genotype selection and rootstock choice are among the most effective long-term strategies for mitigating climate-induced reductions in vegetative growth [78]. Rootstocks regulate root architecture, hydraulic conductivity, water-use efficiency, nutrient acquisition, and hormone transport, thereby improving tree performance under drought and heat stress [89]. Drought-tolerant rootstocks maintain higher stomatal conductance, photosynthetic activity, and antioxidant capacity, while vigorous root systems improve water extraction from deeper soil layers [72]. Scion genotype also determines carbon partitioning and growth responses under elevated CO_2_ and temperature [231]. Consequently, selecting compatible rootstock–scion combinations has become a key breeding objective for maintaining orchard productivity under future climatic conditions.

### Impact of climate change on fruit tree phenology

Fruit trees, as long-lived perennial species, are highly vulnerable to interannual climate variability, making phenology one of the most sensitive indicators of climate change [21]. Phenological events including budburst, flowering, pollination, fruit set, maturation, and leaf senescence are regulated by environmental factors, particularly temperature and atmospheric CO_2_ [21]. Rising temperatures and elevated CO_2_ modify chilling accumulation, dormancy release, and flowering time, leading to changes in fruit yield and quality across many fruit species [18]. Maintaining sustainable fruit production therefore requires a clear understanding of physiological processes such as photosynthesis, respiration, transpiration, hormonal regulation, enzyme activity, membrane stability, and secondary metabolism, which collectively determine tree growth, stress tolerance, and productivity [186]. Although most physiological processes function within a temperature range of approximately 0–40 °C, thermal tolerance varies with genotype, developmental stage, tree age, and stress duration [186]. Heat stress is particularly damaging when it coincides with sensitive phenological stages such as flowering and fruit set [140]. Numerous studies have demonstrated that increasing climate-related stresses reduce fruit yield and quality while altering the geographical suitability of production systems [158]. Consequently, bioclimatic indices and phenological models have become valuable tools for predicting future production zones and assessing orchard adaptation under changing climatic conditions [142]. These models project substantial shifts in spring phenology, chilling accumulation, and the distribution of fruit-growing regions across latitudes and elevations [178]. In addition, climate change disrupts phenological synchrony and increases pest and disease pressure, further threatening orchard productivity and fruit quality [21]. Genetic variation among cultivars and rootstocks strongly influences phenological responses to climate warming [48]. Recent research indicates that rootstocks can modify flowering time, vegetative growth, and fruit maturation through hormonal regulation and altered carbohydrate allocation [84]. Scion genotype determines sensitivity to temperature fluctuations, whereas rootstocks can partially buffer environmental stress by improving water status and nutrient uptake [89]. These genotype-dependent interactions provide opportunities to synchronize flowering and fruit development with future climatic conditions, reducing production risks associated with increasingly variable spring temperatures [35].

### Impact of climate change on bud dormancy

The timing of fruit tree phenology is regulated primarily by air temperature, while photoperiod provides a stable seasonal signal because it is unaffected by climate change [212]. Consequently, rising temperatures have advanced spring phenophases and modified autumn senescence in many fruit species [160]. In temperate deciduous fruit trees, declining temperatures trigger leaf abscission and the onset of dormancy, an adaptive mechanism that protects vegetative and reproductive buds during winter and allows growth to resume only after chilling and heat requirements have been fulfilled [178]. Because dormancy largely determines flowering and fruit set, climate warming has raised concerns about the long-term suitability of traditional fruit-growing regions and intensified research on dormancy regulation and climate adaptation [63]. Bud dormancy comprises three successive phases: para-dormancy, endo-dormancy, and eco-dormancy. Para-dormancy is controlled by inhibitory signals from other plant organs, endo-dormancy by internal physiological mechanisms within the bud, and eco-dormancy by unfavorable environmental conditions, with partial overlap often occurring between the latter two phases [68]. Budbreak requires the fulfillment of cultivar-specific chilling requirements followed by sufficient heat accumulation [70]. This transition is accompanied by hormonal and metabolic changes, including increases in auxins, gibberellins, and cytokinins, reductions in abscisic acid, and alterations in carbohydrate metabolism, enzyme activity, oxidative status, and nutrient reserves, ultimately promoting bud burst and flowering [68]. Because chilling requirements differ substantially among cultivars, they are fundamental for cultivar selection, regional adaptation, and orchard productivity under changing climatic conditions [178]. Successful fruit production therefore depends on the synchronization of winter chilling, spring heat accumulation, and an adequately long growing season to ensure normal flowering, fruit development, and ripening. Bud dormancy regulation is increasingly recognized as a genotype-dependent trait influenced by both scion and rootstock genetics [65]. Recent transcriptomic and physiological studies have shown that rootstocks regulate dormancy-associated genes through hormonal signaling involving ABA, gibberellins, cytokinins, and sugar metabolism [19]. Rootstock-mediated changes in carbohydrate reserves and hydraulic status can accelerate or delay dormancy release depending on environmental conditions [161]. These findings highlight the importance of selecting appropriate rootstock–scion combinations for maintaining stable dormancy cycles under warmer winters [31].

### Impact of climate change on chilling requirement

The chilling requirement is the cumulative exposure to low temperatures required for the release of bud dormancy and initiate normal spring growth [91]. Winter chill is therefore a key determinant of regional suitability for temperate fruit production, particularly in areas with mild winters. Where chilling is inadequate, successful production depends on low-chill cultivars and dormancy-breaking treatments, although accurate estimation remains difficult because budbreak also requires sufficient heat accumulation [61]. The importance of chilling has been demonstrated in numerous temperate fruit species, including apple, pear, apricot, almond, cherry, and pomegranate [149]. In most deciduous fruit trees, effective chilling occurs between 0 and 7 °C, after which spring warming promotes bud burst and flowering [118]. Climate change is disrupting winter chill accumulation and altering phenological development worldwide. Warmer winters have advanced flowering in some regions while reducing chill accumulation in others, resulting in delayed or uneven budbreak, poor flowering synchrony, reduced pollen viability, lower fruit set, and diminished fruit quality [61]. Insufficient chilling has already caused serious production losses, including pear barrenness in subtropical India and reduced fruit set, coloration, storage life, and increased pest incidence in apple [237]. Chilling deficiency also delays leaf emergence and reduces overall yield and fruit quality [193]. Consequently, several phenological models have been developed to estimate cultivar-specific chilling requirements and predict future production suitability under changing climates [57]. Projected climate warming is expected to reduce winter chill across many traditional fruit-growing regions, leading to irregular flowering, bud abortion, poor pollination, fruit abnormalities, and lower orchard profitability [157]. Although low-chill cultivars provide a practical adaptation strategy, they may become more vulnerable to false-spring events and late frost damage [157]. Budbreak also depends on adequate heat accumulation, commonly expressed as growing degree hours (GDH), emphasizing that both chilling fulfillment and spring warming must be considered when evaluating orchard suitability [148]. Climate change further disrupts pollination by reducing flowering overlap among cultivars and pollinizers, particularly in cross-pollinated fruit species such as apple, pear, and walnut, while unfavorable weather during bloom reduces fertilization success in wind-pollinated crops including walnut, pistachio, and hazelnut [179]. Similar effects have been observed in kiwifruit and olive, where insufficient winter chilling has substantially reduced flower bud differentiation, flowering, and yield, highlighting the critical importance of adequate chilling for sustainable fruit production under future climate scenarios [179]. Reduced winter chilling has become a major constraint for temperate fruit production,however, substantial genetic variability exists among cultivars and rootstocks [111]. Recent breeding programs increasingly combine low-chill cultivars with adaptive rootstocks capable of improving flowering uniformity under insufficient chilling conditions [108]. Rootstocks influence heat accumulation, budbreak uniformity, and flowering synchronization by modifying hormonal balance and carbohydrate transport [201]. Integrating low-chill genotypes with climate-resilient rootstocks therefore represents one of the most promising strategies for sustaining fruit production in regions experiencing warmer winters [10].

### Impact of climate change on pollination

Successful fruit production in many species depends on efficient cross-pollination mediated by insects, particularly honeybees, or by wind [99]. Climate change is increasingly disrupting both pollination pathways. High temperatures and adverse weather during flowering reduce pollinator activity, pollen viability, and stigma receptivity, while heavy rainfall limits insect foraging and removes pollen, resulting in poor fertilization, reduced fruit set, and lower yields [87]. Climate-induced reductions in nectar production may further decrease pollinator visitation and pollination success [12]. Heat stress during flowering has been associated with reproductive failure in several fruit crops. In olive, elevated temperatures reduce fruit retention through lower pollen viability and stigma desiccation, whereas in papaya they induce floral sterility, increase flower abscission, and alter floral sex expression [87]. In temperate fruit trees, insufficient winter chilling disrupts flowering synchrony, particularly in wind-pollinated species such as walnut and pistachio, where overlap between male and female bloom is essential for fertilization. Heat stress during floral differentiation may also induce floral abnormalities, especially in Prunus species, leading to malformed fruits and yield reductions [140]. Pollination failure commonly results in poor fertilization, excessive fruit drop, and smaller fruits, as reported in pome fruits, stone fruits, and kiwifruit. In contrast, many tropical and subtropical species, including mango, guava, and lychee, are highly sensitive to low temperatures during flowering, which frequently cause severe flower abscission [186]. Climate warming may also create phenological mismatches between flowering and pollinator emergence, particularly where flowering advances more rapidly than pollinator activity. This asynchrony reduces pollination efficiency and ultimately limits fruit set and orchard productivity [97].

Climate change threatens pollination through altered flowering phenology and reduced synchrony between flowers and pollinators [73]. Recent evidence indicates that genotype selection can partially overcome these challenges by maintaining more stable flowering periods and improved pollen viability under heat stress [131]. Rootstocks also indirectly influence reproductive success by improving tree water relations, flower development, nectar production, and carbohydrate availability during bloom [153]. Consequently, optimizing rootstock–scion compatibility may improve fertilization efficiency and fruit set under increasingly variable climatic conditions.

### Impact of climate change on fruit growth and quality

**Fig. 5:**
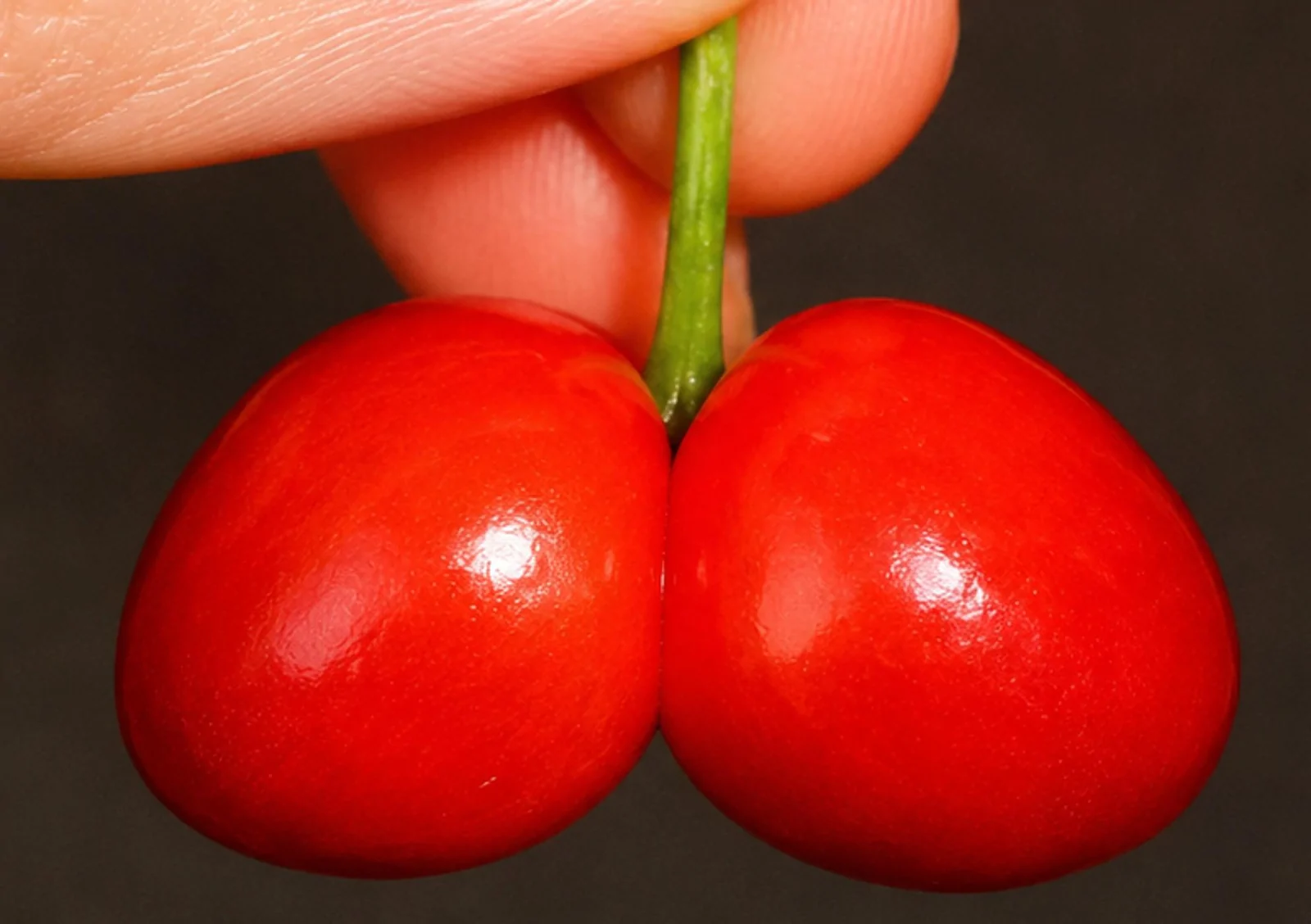
Heat-associated floral bud differentiation disorders expressed as twin (double) sweet cherry fruit formation, linked to elevated temperatures during the preceding summer (taken from [179], with the author's permission)

**Fig. 6:**
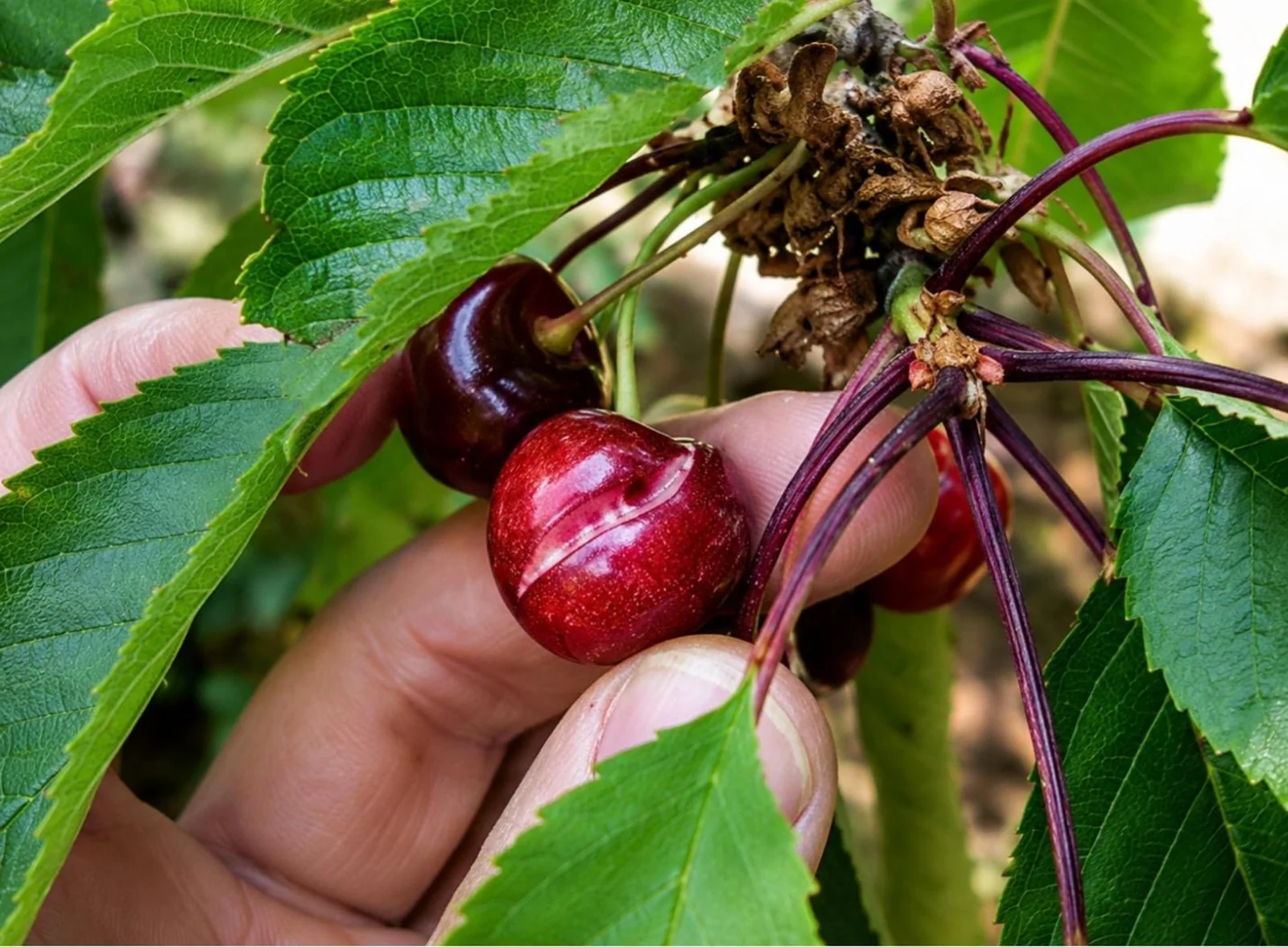
Rain-induced sweet cherry fruit cracking occurring near harvest at full ripeness, illustrating quality losses associated with late-season precipitation events (taken from [179], with the author's permission)

**Fig. 7:**
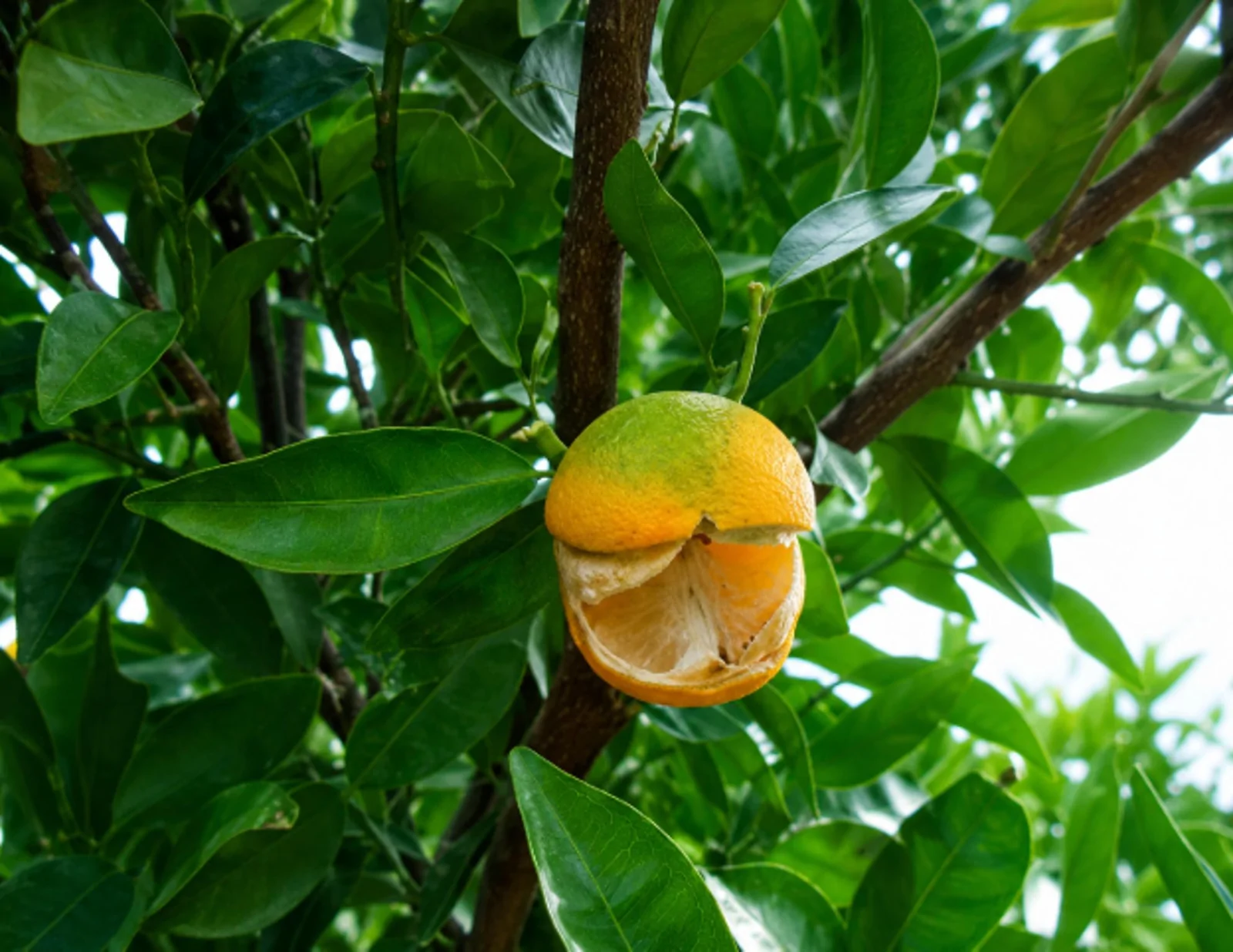
Cracking in orange fruit attributed to irregular irrigation following heavy rainfall, demonstrating the role of abrupt water status fluctuations on peel integrity (taken from [99], with the author's permission)

Projected warming of approximately 0.7–1 °C is expected to alter fruit tree physiology by disrupting photosynthesis, respiration, and carbon metabolism, ultimately affecting fruit yield and quality [53]. Heat stress limits photosynthesis through stomatal closure, reduced CO_2_ diffusion, impaired Rubisco activity, and damage to photosystem II, while simultaneously increasing reactive oxygen species (ROS) and oxidative stress [49]. These physiological disturbances modify primary and secondary metabolism, influencing fruit nutritional composition, flavor, and market quality [140]. Although elevated temperatures have increased antioxidant and flavonoid concentrations in strawberry, they generally reduce fruit quality in crops such as apple, citrus, fig, and pomegranate, emphasizing the need for climate-resilient cultivars [200]. Heat and drought frequently occur together, intensifying their effects on fruit development. Water deficits during cell division and fruit enlargement reduce fruit size, increase fruit drop, and impair sensory and nutritional quality, although moderate stress may enhance the accumulation of phenolic compounds [99, 100]. Excessive solar radiation and high temperatures also induce physiological disorders such as sunburn in apple and citrus, spongy tissue in mango, and floral bud abnormalities that contribute to double-fruit formation in sweet cherry (Fig. 5), reducing both yield and marketability [200]. Altered precipitation patterns further influence fruit quality. Rainfall during late fruit development promotes cracking in sweet cherry (Fig. 6) and pomegranate, rind disorders in citrus (Fig. 7), fruit softening, and reduced postharvest quality, whereas rainfall during early development can enhance shoot growth and nutrient transport [87]. However, warm and humid conditions following rainfall often increase pest and disease incidence [167]. Drought also modifies fruit composition,for example, it reduces the oleic-to-linoleic acid ratio in almond and decreases oil yield while altering phenolic and fatty acid profiles in olive [53]. Not all climate-related effects are negative. Earlier snowmelt at higher latitudes may advance budbreak, extend the growing season, and expand cultivation into previously unsuitable regions [97]. Likewise, elevated atmospheric CO_2_ can enhance photosynthesis, biomass accumulation, and yield when water and nutrient availability are adequate [186], while earlier fruit ripening may provide economic benefits through earlier market access [13]. Nevertheless, climate change substantially alters fruit quality traits, including soluble solids, acidity, color, firmness, flavor, and nutritional composition. High temperatures and intense solar radiation reduce fruit quality in mango, guava, and banana, whereas warm nights suppress anthocyanin accumulation and diminish red skin coloration. Heavy rainfall lowers soluble solids and firmness while increasing fruit cracking and fruit drop, whereas low humidity combined with cool temperatures may promote russeting and delay fruit maturation [104]. Fruit quality responses to climate change are strongly genotype-dependent. Recent studies demonstrate that drought- and heat-tolerant rootstocks improve fruit size, firmness, soluble solids, antioxidant activity, and mineral composition by enhancing water transport and nutrient uptake [197]. Rootstock-mediated regulation of phytohormones and source–sink relationships also contribute to maintaining fruit development under abiotic stress [223]. Therefore, combining superior scion cultivars with climate-resilient rootstocks represents an effective strategy for preserving fruit quality while reducing irrigation requirements under future climates [106].

### Impact of climate change on the prevalence of pests, diseases, and weeds

Climate change is reshaping host–pest–pathogen interactions by altering pest development, survival, reproduction, and geographic distribution while simultaneously affecting host resistance and pathogen virulence [218]. Warmer temperatures and milder winters are expected to increase pest generations, enhance overwintering survival, and facilitate the establishment of invasive species in newly suitable regions, increasing the complexity of crop protection [103]. Global trade further accelerates the spread of invasive organisms. For example, Aleurocanthus spiniferus has expanded from Southeast Asia into the Mediterranean, threatening citrus and grape production, while the olive fruit fly (Bactrocera oleae) is predicted to expand northward under warmer climatic conditions [194]. Similarly, warmer and wetter conditions have intensified olive anthracnose outbreaks, and the bacterial canker pathogen Pseudomonas syringae pv. actinidiae continues to expand in kiwifruit-growing regions [147]. Climate change also favors invasive weeds by altering species composition, extending growing seasons, and increasing reproductive success. Stress-tolerant species such as Ailanthus altissima and Solanum elaeagnifolium are expected to become increasingly dominant, intensifying competition with fruit trees and reducing the effectiveness of conventional weed management practices [209]. Consequently, more adaptive and integrated weed management strategies will be required under future climates [169].

#### Impact of climate change on pest population dynamics

Insect populations are highly sensitive to climatic conditions because temperature directly regulates metabolism, development, reproduction, and dispersal [162]. Rising temperatures generally accelerate insect life cycles, whereas altered precipitation modifies survival and distribution [194]. Heavy rainfall can suppress populations of aphids, mites, whiteflies, and other small insects by washing away immature stages and restricting flight activity, while drought often increases host susceptibility to insect attack [234]. Many economically important pests exhibit species-specific thermal optima. Thrips reproduce most efficiently at 25–30 °C, fruit fly development accelerates between 20 and 35 °C, aphid transmission of Banana bunchy top virus peaks near 25–30 °C, and whitefly populations increase under warm, humid conditions [159]. Climate warming is therefore expected to increase pest pressure in many fruit production systems, although some species may decline where temperatures exceed their physiological limits.

#### Impact of climate change on fruit tree diseases

Climate change strongly influences disease development by modifying interactions among hosts, pathogens, and the environment. Temperature, humidity, rainfall, and solar radiation regulate pathogen survival, infection efficiency, and host susceptibility, while changing climatic conditions may introduce new diseases or intensify existing epidemics [45].

##### Fungal diseases

Fungal pathogens are among the most climate-responsive disease agents. Warmer winters and humid conditions enhance survival and infection of diseases such as anthracnose, powdery mildew, downy mildew, and Phytophthora blight [51]. Anthracnose severity increases under warm, rainy conditions that promote spore dispersal, causing serious losses in mango through flower infection, fruit drop, and postharvest decay [206]. Conversely, prolonged drought and extreme heat may suppress some pathogens while favoring others such as Fusarium and Verticillium, illustrating the species-specific nature of disease responses to climate change [155].

##### Bacterial diseases

Bacterial diseases are closely associated with rainfall and humidity, which facilitate pathogen dispersal and infection. In pomegranate, bacterial blight caused by Xanthomonas axonopodis pv. punicae develops rapidly under high temperatures combined with intermittent rainfall and moderate humidity, resulting in severe fruit cracking and yield losses [121]. Similar climate-dependent patterns have been reported for several bacterial diseases of fruit trees.

##### Viral diseases

The incidence of viral diseases is indirectly influenced by climate through effects on insect vectors. Rising temperatures generally increase populations of aphids, mealybugs, and other vectors, thereby enhancing virus transmission. Pineapple mealybug wilt viruses and banana streak virus are spread by mealybugs, whereas papaya ringspot virus is transmitted by aphids and is most prevalent under warm conditions [134]. Continued warming is therefore expected to expand the distribution and impact of vector-borne viral diseases in fruit production systems.

Genetic resistance is becoming increasingly important as climate change alters pest and pathogen dynamics [93]. Modern rootstocks contribute to integrated pest and disease management by improving root health, enhancing nutrient balance, and activating systemic defense pathways [16]. Recent studies have shown that rootstocks influence expression of defense-related genes, antioxidant metabolism, and microbial recruitment in the rhizosphere, thereby increasing tolerance to soil-borne pathogens and certain insect pests [222]. Future breeding programs are therefore increasingly focusing on rootstock–scion combinations that combine abiotic stress tolerance with durable resistance to emerging pests and diseases, providing a sustainable approach to climate-resilient orchard management.

## Genotype- and rootstock-mediated adaptation to climate change in fruit trees

Climate change is increasingly threatening fruit production by intensifying drought, heat waves, salinity, and inadequate winter chilling, all of which reduce orchard productivity and sustainability [44]. Because fruit trees are perennial crops with long productive lifespans, their capacity to adapt rapidly to changing environments is limited, making genetic improvement and rootstock selection essential adaptation strategies [207]. Considerable variation among cultivars and rootstocks in physiological, biochemical, and molecular stress responses provides valuable opportunities to enhance climate resilience [55]. Beyond controlling tree vigor, rootstocks regulate water and nutrient uptake, phenological development, and long-distance stress signaling between roots and shoots [208]. Moreover, rootstock–scion interactions largely determine tree growth, productivity, and stress tolerance under future climatic conditions [25]. Consequently, understanding genotype- and rootstock-mediated adaptation has become a research priority for developing resilient orchard systems [52]. This review examines recent advances in: (i genetic diversity and cultivar adaptation,(ii rootstock-mediated regulation of water relations and drought tolerance; (iii rootstock effects on salinity tolerance; (iv rootstock-mediated enhancement of physiological and biochemical traits under abiotic stress; (v rootstock effects on phenology and adaptation to altered chilling requirements; (vi rootstock-mediated nutrient uptake and use efficiency; (vii rootstock effects on canopy architecture, tree vigor, and heat avoidance; (viii rootstock–scion interactions under elevated atmospheric CO_2_; and (ix) future perspectives for breeding climate-resilient rootstocks.

### Genetic variability and cultivar adaptation

Genetic diversity is fundamental to climate adaptation in perennial fruit crops because it provides the variability required for selecting and breeding stress-resilient cultivars [156]. Traditional cultivars, landraces, and wild relatives represent valuable reservoirs of adaptive traits for tolerance to drought, heat, salinity, and insufficient winter chilling, making them essential genetic resources for climate-resilient breeding [192]. Considerable genotypic variation has been reported in photosynthetic efficiency, stomatal regulation, water-use efficiency, and root system architecture, all of which determine plant performance under environmental stress [164]. Cultivars with greater carbon assimilation, efficient water uptake, and deeper root systems generally exhibit superior productivity under warming climates [143]. Phenological plasticity further enhances climate adaptation by allowing cultivars to avoid environmental hazards. Late-flowering genotypes can escape spring frosts, whereas low-chill cultivars maintain productivity in regions experiencing warmer winters [46]. Genotypic differences in pollen viability, flowering stability, and fruit set also contribute to reproductive success under heat stress [131]. Recent advances in genomics have accelerated the identification of quantitative trait loci (QTLs), adaptive alleles, and stress-related genes controlling drought tolerance, phenology, and heat adaptation in major fruit crops [238]. Marker-assisted and genomic selection have substantially improved breeding efficiency by enabling rapid selection of superior genotypes carrying multiple stress-resilience traits [122]. Recent studies also highlight the importance of genotype-specific regulation of antioxidant systems, osmotic adjustment, hormonal signaling, and epigenetic modifications in long-term climate adaptation [233]. Integrating genomics, transcriptomics, phenomics, and high-throughput phenotyping with multi-environment field evaluations is improving the identification of climate-resilient cultivars while accounting for strong genotype × environment interactions [14]. Consequently, modern breeding programs are increasingly shifting from maximizing yield under optimal conditions toward developing cultivars that combine stable productivity, stress resilience, and superior fruit quality under future climate scenarios [232].

### Rootstock-mediated regulation of water relations and drought tolerance

Water scarcity is becoming one of the greatest challenges for sustainable fruit production under climate change, making drought-tolerant rootstocks an essential component of climate-resilient orchards [11]. Rootstocks regulate whole-tree water relations by influencing root architecture, hydraulic conductivity, and root-to-shoot signaling, thereby determining the drought tolerance of grafted trees [210]. Rootstocks with deep, highly branched root systems improve access to soil moisture, enhance water uptake efficiency, and sustain tree growth during prolonged drought [43]. Hydraulic efficiency is equally important, as drought-tolerant rootstocks possess xylem characteristics that reduce cavitation and embolism, preserving water transport, transpiration, and photosynthetic activity under water deficit [204]. Rootstock-mediated chemical signaling further enhances drought adaptation. Abscisic acid (ABA) synthesized in drought-stressed roots regulates stomatal closure, reducing transpirational water loss and improving water-use efficiency, while differences in ABA signaling largely explain the contrasting drought responses among graft combinations [55]. In addition, aquaporins facilitate water transport across root membranes, and recent transcriptomic studies indicate that drought-tolerant rootstocks exhibit distinct aquaporin expression patterns associated with improved hydraulic performance [54]. Osmotic adjustment through proline, soluble sugars, and polyols also contributes to maintaining cell turgor and metabolic activity under drought stress [187]. Rootstocks strongly influence scion physiology by maintaining higher leaf water potential, relative water content, stomatal conductance, and photosynthetic capacity during water deficits, thereby improving yield stability [230]. They also modify canopy architecture, with dwarfing rootstocks reducing canopy size and transpirational demand, leading to greater water-use efficiency [89]. Significant differences in drought tolerance have been reported among commercial rootstocks for apple, citrus, grapevine, peach, almond, and sweet cherry, particularly those derived from xerophytic species [50, 140, 243]. Recent studies further demonstrate that drought tolerance depends on interactions among rootstocks, antioxidant defense systems, and the rhizosphere microbiome. Rootstocks that enhance superoxide dismutase, catalase, and peroxidase activities better protect grafted trees from oxidative damage, while associations with arbuscular mycorrhizal fungi and beneficial rhizosphere microorganisms improve water uptake, nutrient acquisition, and stress resilience [33, 74]. Consequently, integrating drought-tolerant rootstocks with deficit irrigation and precision water management has emerged as one of the most effective strategies for sustaining orchard productivity under increasing water scarcity [75, 138].

### Rootstock effects on salinity tolerance

Soil salinization is becoming an increasingly serious constraint to fruit production owing to climate change, declining freshwater resources, and intensive irrigation practices [113, 202]. Rootstocks play a decisive role in determining the salinity tolerance of grafted fruit trees, with substantial genotypic variation in their capacity to exclude toxic ions and maintain tree productivity [15, 221]. Salt-tolerant rootstocks restrict the uptake and translocation of Na⁺ and Cl⁻ to the scion, thereby preserving chlorophyll content, photosynthetic efficiency, and leaf function [37, 86]. Their ability to maintain favorable K⁺/Na⁺ and Ca^2^⁺/Na⁺ ratios is fundamental for sustaining cellular homeostasis and metabolic activity under saline conditions [26]. These processes are regulated by membrane transporters, including HKT, SOS, and NHX proteins, which control ion transport and vacuolar sequestration, minimizing cytoplasmic toxicity [213, 241]. Besides ion homeostasis, osmotic adjustment is a major mechanism of salinity tolerance. Salt-tolerant rootstocks accumulate compatible solutes such as proline, soluble sugars, and glycine betaine, which maintain cell turgor, stabilize membranes, and protect cellular metabolism [41]. Because salinity also promotes excessive reactive oxygen species (ROS) production, efficient antioxidant defense systems are essential. Rootstocks exhibiting higher activities of superoxide dismutase, catalase, and peroxidase show reduced oxidative damage and improved stress tolerance in pear, grapevine, citrus, and stone fruit species [66, 146, 221]. Accordingly, rootstock genotype largely determines leaf injury, growth reduction, and tree survival under saline conditions [247]. Substantial differences in salinity tolerance have been reported among commercial rootstocks of citrus, grapevine, pistachio, almond, peach, and olive [5, 20, 92, 175, 220]. Structural modifications, including increased root suberization and lignification, further restrict passive Na⁺ uptake, while root exudates and associations with arbuscular mycorrhizal fungi improve nutrient acquisition and rhizosphere function under saline conditions [114, 216, 219]. Rootstocks also regulate hormonal signaling through abscisic acid and ethylene, enhancing physiological adaptation to salinity [136]. As drought and salinity frequently occur simultaneously in orchard systems, breeding efforts increasingly target rootstocks with combined tolerance to multiple abiotic stresses [227]. Recent transcriptomic and multi-omics studies have identified key genes involved in ion transport, osmotic adjustment, antioxidant metabolism, and stress signaling, providing valuable tools for accelerating rootstock improvement [146, 165]. Therefore, integrating salt-tolerant rootstocks with precision irrigation and improved soil management has become a cornerstone of climate-resilient orchard production, while future breeding programs should prioritize rootstocks capable of simultaneously tolerating salinity, drought, and heat stress [154, 156].

### Rootstock-mediated enhancement of physiological and biochemical traits in fruit trees under abiotic stress

Recent research has highlighted the central role of rootstocks in regulating the physiological and biochemical responses of grafted fruit trees to environmental stresses, making rootstock selection a key strategy for climate-resilient orchard management [9, 190]. Stress-tolerant rootstocks enhance photosynthetic performance by improving water uptake, hydraulic conductivity, and stomatal regulation, enabling scions to sustain higher carbon assimilation under drought, salinity, and heat stress [56, 116, 171]. They also preserve chlorophyll content and improve chlorophyll fluorescence parameters, including Fv/Fm and ΦPSII, thereby maintaining photosystem II stability, reducing photoinhibition, and protecting the photosynthetic apparatus from oxidative damage [30, 242]. Consequently, grafted trees maintain greater photosynthetic efficiency and productivity under adverse conditions. Improved photosynthesis promotes carbohydrate production and assimilate transport to developing fruits. Accordingly, stress-tolerant rootstocks increase the accumulation of soluble sugars, which function not only as energy reserves but also as osmoprotectants that stabilize membranes, maintain cell turgor, and support metabolic activity under drought and salinity stresses [2, 133, 140]. Increased soluble protein content has also been reported in grafted trees, reflecting enhanced synthesis of heat shock proteins, molecular chaperones, and other stress-responsive proteins that preserve cellular homeostasis [215, 228, 229]. Likewise, higher proline accumulation in scions grafted onto tolerant rootstocks contributes to osmotic adjustment, enzyme protection, and reactive oxygen species (ROS scavenging, reinforcing stress adaptation [102]. Rootstocks further strengthen abiotic stress tolerance by activating antioxidant defense systems. Increased activities of superoxide dismutase, catalase, peroxidase, and ascorbate peroxidase reduce ROS accumulation, limit lipid peroxidation, preserve membrane integrity, and delay stress-induced senescence [124, 189]. Recent transcriptomic, metabolomic, and multi-omics studies reveal that these responses are coordinated through rootstock–scion signaling networks involving abscisic acid, cytokinins, jasmonates, and salicylic acid, which regulate photosynthesis, osmotic adjustment, antioxidant metabolism, and stress-responsive gene expression [9, 84, 203]. Collectively, these findings demonstrate that selecting appropriate rootstocks is a climate-smart approach for sustaining fruit yield and quality, while future breeding programs should integrate physiological, biochemical, and molecular traits to develop next-generation rootstocks with broad tolerance to multiple abiotic stresses [207].

### Rootstock influence on phenology and chilling requirement adaptation

Climate warming is substantially altering the phenology of fruit trees by reducing winter chill accumulation and advancing spring development, making insufficient chilling a major limitation in many temperate fruit-growing regions [87, 166]. Under these conditions, rootstocks have become important regulators of scion phenology and valuable tools for improving orchard adaptation to climate change [76]. Numerous studies demonstrate that rootstocks influence budbreak, flowering, fruit set, and fruit maturation, thereby helping synchronize tree development with local environmental conditions [35, 201]. Rootstocks that delay flowering can reduce the risk of late spring frost damage, whereas those promoting earlier budbreak are advantageous in regions experiencing inadequate winter chilling [141, 183]. Significant rootstock-dependent variation in flowering time has been reported in apple, peach, sweet cherry, apricot, almond, and citrus [120, 183]. The regulation of phenology is largely mediated through rootstock-induced changes in vigor, hormonal signaling, carbohydrate dynamics, and tree water relations. Vigorous rootstocks generally delay reproductive development compared with dwarfing rootstocks [226]. Root-derived phytohormones, particularly cytokinins, gibberellins, and abscisic acid, regulate dormancy induction, maintenance, and release, while carbohydrate storage and remobilization support uniform budbreak, flowering, and early shoot growth [90, 101]. Rootstocks also influence hydraulic conductivity, bud hydration, canopy architecture, and nutrient uptake, all of which indirectly affect phenological progression and reproductive development [24, 34, 35]. Recent molecular studies further demonstrate that rootstocks modify the expression of key dormancy- and flowering-related genes, including DAM, FT, SVP, and CBF, providing a mechanistic basis for rootstock-mediated phenological regulation [65, 80, 190]. These interactions can partially compensate for inadequate winter chilling by improving budbreak uniformity, flowering intensity, fruit set, and overall productivity in low-chill environments [96, 108]. Because phenological responses arise from complex rootstock × scion × environment interactions, multi-year and multi-location evaluations remain essential for identifying stable combinations [181, 199]. As winter chill is projected to decline further, selecting phenology-modifying rootstocks will become increasingly important for sustainable fruit production [156]. Rootstocks that delay bloom while maintaining fruit set are particularly valuable in frost-prone regions, whereas those promoting early and uniform budbreak can improve productivity in warmer climates with insufficient chilling [67, 183]. Future breeding programs should integrate low-chill cultivars with climate-adapted rootstocks and exploit advances in molecular biology to better understand root-to-shoot signaling networks controlling dormancy and flowering. Such integrated strategies will accelerate the development of resilient orchard systems capable of maintaining stable productivity under future climate scenarios [47].

### Rootstock-mediated nutrient uptake and use efficiency

Efficient nutrient acquisition and utilization are essential for sustaining fruit tree productivity under climate change, as rising temperatures, erratic rainfall, drought, and soil degradation increasingly limit nutrient availability in orchard systems [22, 196]. In grafted fruit trees, rootstocks play a central role in regulating nutrient uptake, transport, and utilization, with considerable genotypic variation in nutrient-use efficiency under both optimal and stressful conditions [40, 214]. Rootstocks with deep, highly branched root systems and greater root surface area generally exhibit superior nutrient acquisition, while enhanced root hair development further improves rhizosphere exploration and nutrient absorption [21, 211]. Significant differences among rootstocks have been reported in the uptake of both macronutrients (N, P, K, Ca, Mg, and S) and micronutrients (Fe, Zn, Mn, B, and Cu), directly affecting tree vigor, yield, and fruit quality [77, 236]. Rootstock-mediated nutrient efficiency becomes particularly important under drought because limited soil moisture restricts nutrient mobility. Drought-tolerant rootstocks maintain higher nutrient uptake and utilization by improving root hydraulic function and rhizosphere activity [40, 72]. Root exudates, including organic acids and protons, increase nutrient solubility, particularly phosphorus and micronutrients, while beneficial interactions with arbuscular mycorrhizal fungi and plant growth-promoting rhizobacteria further enhance nutrient acquisition and stress tolerance [125, 216, 225, 235]. Rootstocks also regulate xylem transport, improving calcium translocation and reducing physiological disorders such as bitter pit, blossom-end rot, and fruit cracking [83]. Beyond nutrient uptake, rootstocks influence nitrogen metabolism and overall nutrient-use efficiency, thereby supporting photosynthesis, biomass production, and fruit quality while reducing fertilizer requirements [39, 205]. Because nutrient efficiency is closely linked with drought and salinity tolerance, future climate-resilient rootstocks should combine efficient nutrient acquisition with broad-spectrum abiotic stress resistance [151]. Recent transcriptomic studies have identified genotype-specific expression of nitrate, phosphate, potassium, and iron transporter genes, providing new insights into the molecular regulation of nutrient uptake [6, 244]. Advances in genomics, phenomics, and multi-omics, together with precision fertilization strategies, are accelerating the development of nutrient-efficient rootstocks and optimizing fertilizer management for different graft combinations [36, 71]. Integrating these rootstocks with sustainable orchard management will be critical for improving resource-use efficiency, reducing environmental impacts, and maintaining fruit production under future climate scenarios.

### Rootstock effects on canopy architecture, vigor, and heat avoidance

Rootstocks play a fundamental role in determining canopy architecture and vegetative vigor, thereby influencing the capacity of fruit trees to adapt to climate change [207]. Canopy structure regulates light interception, canopy microclimate, transpiration, and fruit exposure to environmental stress, making it a key determinant of orchard performance under warming conditions [35, 38]. Dwarfing rootstocks are widely used in high-density orchards because they reduce vegetative growth, improve land- and water-use efficiency, and facilitate orchard management [182]. However, excessive dwarfing may increase fruit exposure to solar radiation and the risk of sunburn, whereas highly vigorous rootstocks provide greater canopy shading but generally increase transpiration and water demand. Consequently, moderately vigorous rootstocks often provide the best balance between productivity, water conservation, and heat protection [144, 168]. Rootstocks also modify branch angle, shoot growth, internode length, canopy density, and leaf morphology, thereby influencing canopy porosity, light distribution, and temperature regulation [24, 245]. While dense canopies reduce fruit surface temperature through shading, excessive canopy closure may limit light penetration, impair fruit coloration, and reduce quality. Conversely, open canopies improve air circulation, light interception, and disease management [17, 139]. Rootstocks with high hydraulic conductivity maintain transpiration and stomatal function during heat stress, enhancing evaporative cooling, sustaining photosynthesis, and reducing heat-induced damage [58, 130, 188]. These physiological traits are becoming increasingly important as heat waves intensify across fruit-growing regions. Canopy architecture strongly influences fruit quality by regulating the thermal and light environment surrounding developing fruits. Excessive solar exposure can cause sunburn, poor coloration, skin browning, and changes in fruit biochemical composition, whereas appropriate canopy density protects fruit while maintaining sufficient light for quality development [145, 190]. Significant rootstock effects on canopy temperature and fruit microclimate have been reported in apple, citrus, peach, and grapevine [81, 153]. Therefore, selecting suitable rootstocks should be integrated with pruning, training systems, and canopy management to optimize light interception and heat avoidance [173]. Future breeding efforts should prioritize rootstocks combining moderate vigor, efficient water use, and enhanced heat tolerance, enabling climate-resilient orchard systems capable of maintaining fruit yield and quality under increasingly extreme environmental conditions [8, 44].

### Rootstock–scion interactions under elevated atmospheric CO_2_

Atmospheric carbon dioxide (CO_2_) concentrations are projected to continue increasing throughout the twenty-first century, with major implications for the physiology and productivity of perennial fruit crops [191]. Elevated CO_2_ generally stimulates photosynthesis in C_3_ fruit trees by enhancing carboxylation efficiency while reducing photorespiration [69]. However, the magnitude and persistence of these benefits largely depend on rootstock–scion interactions, which regulate water uptake, nutrient acquisition, hormonal signaling, and sink strength [59, 84]. Consequently, grafted fruit trees exhibit considerable variation in growth and productivity under elevated CO_2_. Rootstocks with strong sink capacity can sustain enhanced photosynthesis, whereas limited sink demand promotes carbohydrate accumulation and photosynthetic downregulation [95, 126]. Such acclimation has been reported in apple, citrus, grapevine, and peach [69, 79]. Rootstock genotype also influences carbon partitioning among roots, shoots, and fruits. Vigorous rootstocks generally enhance vegetative biomass, whereas dwarfing rootstocks favor reproductive development and orchard efficiency [82]. Although elevated CO_2_ often increases yield through greater carbon assimilation, fruit quality responses remain genotype-dependent [85]. Rootstocks that efficiently allocate assimilates to reproductive organs are therefore expected to improve both yield and fruit size under future atmospheric conditions [8]. Elevated CO_2_ also modifies root architecture by increasing root biomass and soil exploration capacity, thereby improving water and nutrient acquisition [69]. A well-established response is reduced stomatal conductance, which decreases transpiration while enhancing intrinsic water-use efficiency [4]. Rootstocks regulate these responses through hydraulic conductivity, aquaporin activity, and abscisic acid signaling [163]. Consequently, rootstocks that maintain favorable plant water status despite lower stomatal conductance are likely to improve drought resilience [116, 240]. Nutrient acquisition represents another key aspect of rootstock-mediated adaptation under elevated CO_2_. Increased carbon assimilation frequently exceeds nutrient uptake, leading to dilution of mineral nutrients, particularly nitrogen [32, 180]. Because rootstocks differ substantially in their capacity to absorb and transport nutrients, they strongly influence scion nutritional status [211]. Rootstocks with greater nutrient-use efficiency can help sustain productivity and fruit quality by improving the uptake of nitrogen, phosphorus, potassium, calcium, and essential micronutrients under elevated CO_2_ [94, 195]. Under field conditions, elevated CO_2_ interacts with heat, drought, and salinity rather than acting independently [3]. Although elevated CO_2_ can partially mitigate drought stress by improving water-use efficiency and maintaining photosynthesis [177], these advantages are often reduced under concurrent high temperatures because of increased respiration and impaired reproductive development [60]. Therefore, rootstocks combining heat and drought tolerance will be increasingly important for sustaining orchard productivity under future climates [185]. Recent studies highlight hormonal and molecular communication between rootstock and scion as central regulators of acclimation to elevated CO_2_. Root-derived hormones including cytokinins, abscisic acid, gibberellins, and strigolactones coordinate shoot growth, stomatal behavior, and source–sink relationships [64]. In addition, mobile RNAs, peptides, proteins, and epigenetic regulators contribute to long-distance signaling across the graft union, altering gene expression and stress-response pathways [172]. Advances in transcriptomics, proteomics, metabolomics, and ionomics are providing new insights into these mechanisms [27], although the molecular regulation of rootstock–scion interactions under future atmospheric conditions remains incompletely understood [138]. Future studies integrating omics, long-term field experiments, and free-air CO_2_ enrichment systems should identify rootstock–scion combinations capable of maintaining productivity, fruit quality, and resource-use efficiency under elevated CO_2_ combined with multiple environmental stresses [184]. Such knowledge will be essential for developing climate-resilient and sustainable orchard systems.

### Future perspectives for breeding climate-resilient rootstocks

Climate change is expected to intensify drought, heat waves, salinity, flooding, and erratic winter temperatures, creating an urgent need for next-generation rootstocks adapted to multiple abiotic stresses [107]. Whereas traditional breeding has focused on vigor control, disease resistance, and graft compatibility, future programs must prioritize resilience to combined environmental stresses while maintaining productivity, fruit quality, and resource-use efficiency [117, 208]. However, conventional breeding in perennial fruit crops is hindered by long juvenile phases and lengthy field evaluations, making advanced breeding technologies increasingly important [132, 170]. Recent advances in molecular genetics and genomics have accelerated rootstock improvement. Marker-assisted selection (MAS) enables early identification of traits such as drought and salinity tolerance, dwarfing, and disease resistance, substantially shortening breeding cycles [23]. Likewise, genomic selection predicts breeding values from genome-wide markers before full phenotypic evaluation, making it especially valuable for perennial fruit crops [29]. High-throughput sequencing has further facilitated the discovery of quantitative trait loci (QTLs) and candidate genes associated with stress adaptation in apple, citrus, grapevine, Prunus species, and nut crops, while continued sequencing of elite rootstocks will accelerate allele discovery and trait improvement [135, 224]. In addition, wild relatives and traditional landraces remain important reservoirs of adaptive alleles for improving tolerance to drought, salinity, and other climate-related stresses [7, 28]. The integration of multi-omics approaches including genomics, transcriptomics, proteomics, metabolomics, and phenomics is providing a comprehensive understanding of the mechanisms underlying rootstock-mediated stress tolerance [146]. Combined with systems biology, these approaches are revealing regulatory networks controlling water relations, nutrient acquisition, hormonal signaling, and stress acclimation [123]. Root-to-shoot communication has emerged as a key research frontier, with hormones, mobile RNAs, peptides, and epigenetic regulators coordinating scion responses to environmental stress [105, 109, 223]. These discoveries provide valuable targets for breeding climate-resilient rootstocks. Gene-editing technologies, particularly CRISPR/Cas systems, are transforming rootstock breeding by enabling precise modification of stress-responsive genes controlling water-use efficiency, salinity exclusion, nutrient uptake, and phenological adaptation [42, 228, 229]. Emerging base-editing and prime-editing technologies further improve editing accuracy while minimizing unintended genomic changes. Nevertheless, successful commercial deployment will depend on supportive regulatory frameworks and public acceptance [129]. Future breeding strategies should increasingly evaluate rootstocks under combined stress conditions rather than single stress factors, reflecting the complexity of field environments [176]. High-throughput phenotyping platforms, using remote sensing, thermal imaging, hyperspectral sensors, and unmanned aerial systems, now enable rapid and non-destructive assessment of plant performance under stress [110]. In parallel, artificial intelligence and machine learning are improving the analysis of complex phenotypic datasets and strengthening predictive breeding approaches. Breeding programs should also prioritize traits related to water-use efficiency, carbon sequestration, nutrient-use efficiency, and beneficial interactions with the rhizosphere microbiome to support sustainable orchard systems [67, 112, 198]. Long-term, multi-location field trials remain essential for assessing genotype × environment interactions across diverse production regions [239]. Collaborative initiatives such as the NC-140 rootstock trials demonstrate the importance of coordinated evaluation for identifying stable and broadly adapted rootstocks [174]. Ultimately, integrating conventional breeding with genomics, multi-omics, gene editing, high-throughput phenotyping, artificial intelligence, and climate modeling will be critical for developing climate-resilient rootstocks capable of sustaining global fruit production under future environmental conditions [228, 229].

## Conclusions

Climate change will increasingly challenge the sustainability of fruit tree production because perennial species experience cumulative effects of heat, drought, salinity, inadequate winter chilling, and other environmental stresses over multiple growing seasons. The evidence synthesized in this review demonstrates that the degree of climate resilience is highly genotype-dependent, with both scion and rootstock characteristics playing central roles in determining physiological performance, phenological stability, water-use efficiency, nutrient acquisition, and yield under adverse conditions. Consequently, future adaptation strategies should prioritize the identification and deployment of genotype-specific rootstock–scion combinations rather than relying on universal management solutions. A major research priority is the systematic evaluation of diverse germplasm and rootstocks under combinations of abiotic stresses that more accurately reflect future orchard environments, since multiple stresses frequently occur simultaneously and elicit responses that differ from those observed under single-stress conditions. Equally important is the validation of promising genotypes through long-term, multi-location orchard trials to assess their stability, productivity, and fruit quality across contrasting climates and production systems. Future progress will depend on integrating physiological phenotyping, molecular genetics, genomics-assisted breeding, and precision orchard management, including optimized irrigation, nutrient management, and environmental monitoring. Such multidisciplinary approaches will accelerate the development of climate-resilient fruit production systems while improving resource-use efficiency and reducing production risks. Collectively, these strategies provide the most robust pathway for sustaining orchard productivity, preserving fruit quality, and maintaining the economic and environmental sustainability of perennial fruit production under future climate scenarios.

## Data Availability

Not applicable. Because this article is a review and no new datasets were generated.
